# Experimental system and method of aerobic thermal environment simulation based on laser heating

**DOI:** 10.1038/s41598-024-67426-7

**Published:** 2024-07-15

**Authors:** Jiawei Wang, Bin Li, Shengwu Li, Sihao Gao, Yanlong Shen, Dahui Wang, Pengling Yang

**Affiliations:** 1 State Key Laboratory of Laser Interaction with Matter, Northwest Institute of Nuclear Technology, Xi’an, 710024 China; 2 Institute of Aeronautics, Northwestern Polytechnical University, Xi’an, 710072 China

**Keywords:** High temperature, Laser, Thermal-oxidation, Flat-topped beam, Carbon matrix composites, Characterization and analytical techniques, Design, synthesis and processing, Aerospace engineering

## Abstract

Considering the superior luminous intensity characteristics of lasers, a thermal simulation platform employing laser-induced heating in an aerobic environment was developed. Achieving a uniformly distributed flat-topped square laser beam output was facilitated through optical fibre bundling techniques, while precise control over laser power output was attained through current modulation. Utilising the aforementioned system, thermal shock simulation experiments were conducted in an aerobic environment, subjecting two types of high-temperature-resistant composites, namely C/C and C/SiC, to temperatures up to 1800 °C. These composites were lightweight, heat-resistant materials designed for hypersonic vehicle applications. The results show that the system and method can be used to simulate high temperatures, rapid temperature increases, and thermal shocks on C/C composite materials, with minimal variation in the coupling coefficient under aerobic conditions. The system and method can also provide key technology support for thermal-force-oxygen coupling testing of high temperature resistant materials.

## Introduction

With the development of high-temperature-resistant materials, particularly carbon-based composites, these materials have found widespread application in aerospace for the thermal protection structures of rocket engines and hypersonic vehicles^1,2^. In order to evaluate the anti-ablation effect of high temperature structures prepared by carbon matrix composites under aerobic and extremely high temperature conditions, surface thermal simulation testing needs to be conducted^3^. According to analysis, the main difficulty in thermal simulation of the aforementioned high-temperature-resistant materials or structures in aerobic and high-temperature environments is the selection of suitable heat sources capable of generating high temperatures or rapid temperature rise rates. Numerous international and domestic scholars have conducted relevant research on this issue. Environment irradiation heating technology, often utilised in such scenarios^4–6^, typically employs graphite or molybdenum wire as heating elements. This method offers benefits such as uniform temperature distribution, low thermal inertia, and straightforward control. However, it suffers from a drawback of relatively slow temperature rise rates, typically in the range of 10 °C per minute. As such, achieving high-temperature environment simulation poses challenges with this approach^7–9^. Both the electric resistance heating method and the induction heating method offer advantages such as high heating temperatures and rapid heating rates. However, a limitation of these methods is that they are primarily suitable for heating conductive materials and are not well-suited for non-conductive materials such as SiC^10–12^. Several scholars have explored the use of quartz lamps, halogen lamps, and similar radiation-based heating methods. Nevertheless, these methods are constrained by the melting temperature of the quartz glass surrounding the quartz lamp heating tube, imposing a limit on the achievable heating temperature. Typically, this limit is around 1600 °C^13–15^. There are challenges in observing the heating morphology of the specimen because quartz lamps can block the surface of the specimen when heated. Arc wind tunnel heating^16^ offers an effective means to simulate diverse aerodynamic heating test environments. However, the substantial cost associated with large-scale wind tunnel experimental equipment, along with high operational and maintenance expenses, restricts its utilisation primarily to experimental research involving large-scale structural components. Oxygen–acetylene flame heating has the advantages of high heating rate and high heating temperature^17^, but challenges arise from the difficulty in precisely controlling the flow velocity of the oxygen–acetylene mixture. This lack of control hampers the accurate regulation of the material heating process.

The laser heating method has a number of advantages, including rapid temperature rise, high maximum heating temperatures, and exceptional versatility^18–20^. In recent years, with the development of laser technology, laser heating has been adopted to measure the melting point of high-temperature resistant materials^21^. Predictably, the high brightness of lasers inherently leads to heating with characteristics of high temperatures and rapid temperature rise rates. Additionally, the exceptional directivity of lasers facilitates unobstructed observation of the heating process without the need for specimen shielding, enabling in-situ measurement of the ablation process of high-temperature resistant materials. Hence, the high-temperature environment simulation of materials based on laser heating has a considerably broad application prospect. In thermal environment simulation, achieving uniform laser intensity irradiating the surface is crucial. Therefore, laser heating requires a laser beam with high uniformity, typically in the form of a flat-topped beam. Several reports have described laser sources capable of producing high homogeneity flat top beam outputs. For instance, a CO_2_ laser heating source has been built in the HEMEL laboratory, which can realise high homogeneity flat top beam output^22–24^. Notably, the wavelength of CO_2_ laser primarily falls around 10 μm. The wavelength of CO_2_ lasers can indeed interfere with surface temperature measurements within the response wavelength range of traditional infrared radiation temperature measurement systems, such as thermal imagers. As a result, there have been no reported cases of CO_2_ lasers being used to simulate high-temperature thermal environments for materials. Consequently, there remains a need to develop a laser beam with high uniformity and a flat-top profile, while also possessing a suitable output wavelength for effectively simulating thermal conditions for carbon matrix composites. Existing research indicates that the output power of single fibre lasers can reach the kilowatt level^25–27^. Further, by combining multiple fibre laser beams, it is possible to achieve high homogeneity and high brightness flat-top laser outputs^28–30^. This advancement opens up the possibility of utilising laser heating for simulating thermal environments in high-temperature-resistant materials. Leveraging high homogeneity and high brightness fibre lasers, thermal environment simulations were conducted on high-temperature-resistant materials. It enabled in-situ observation of material surface morphology under conditions of rapid temperature rise and high-temperature environments. The present study can provide key technology support for evaluating the performance of high-temperature-resistant materials under extreme thermal conditions.

## Experimental and results

### Composition of experimental system

**Figure 1:**
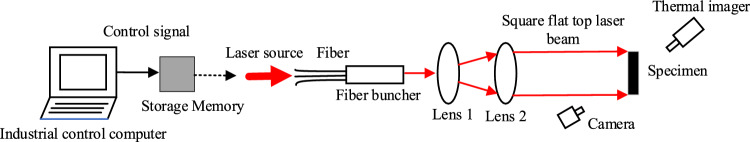
Composition of experimental system of laser heating.

Given the current development level of fibre lasers, which enables flexible control of laser output power through current modulation, the experimental system employs a fibre laser as the heat source to generate high-temperature environments, as depicted in Fig. 1. The system is composed of an industrial control computer, laser unit, memory module, lens apparatus, thermal imager and camera. The industrial control computer is used to adjust the output power of the laser and realise the automatic control of the temperature setting of the start-up heating test. The specific method involves editing the current output signal in the computer and sending it to the memory integrated in the laser source, the laser source receives the real-time change of the current signal instruction, outputs the specific laser power, and irradiates the specimen surface after the lens group is shaped into a square uniform light spot. The thermal imager is utilised to document the temperature rise progression of the specimen surface under laser irradiation, with its response range spanning from 100 to 3000 °C. Meanwhile, the camera serves to monitor alterations in the surface morphology of the specimen throughout the laser heating process.

**Figure 2:**
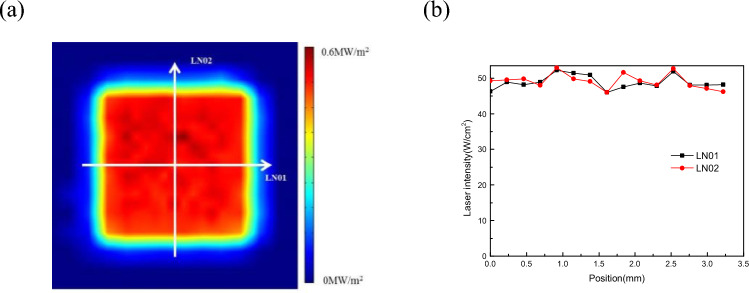
Intensity distribution of laser spot: (**a**) visual results; (**b**) test results of LN01 and LN02.

The output wavelength of the laser is 1080 nm and the maximum heating power is 6000 W. To generate a uniform laser intensity irradiating the surface field, the system achieves a flat-topped square spot output with high homogeneity by employing multi-mode optical fibre bunching techniques. Figure 2a illustrates the visual distribution of the laser spot intensity. To validate the uniformity of the laser intensity distribution, straight lines LN01 and LN02 were drawn at the centre of the transverse and vertical axes, respectively. The power density of characteristic points along these lines was selected to assess the uniformity of the laser spot intensity, as depicted in Fig. 2b. The results indicate minimal variation in the intensity distribution of the laser spot at different locations. Therefore, it can be concluded that the thermal field generated by the laser heating in the proposed experimental system can be considered equivalent to a uniform surface laser intensity irradiating the surface.The laser intensity in Fig. 2 was measured by an array distributed photodetector. The uncertainty of the system is determined by the photodetector, which is approximately 3%(k = 2). The uncertainty of temperature measurement in the article is determined by the infrared thermal imager, which has a temperature measurement uncertainty of about 2%(k = 2).

### High laser intensity irradiating the surface heating experiment in aerobic environment

#### Laser heating C/C COMPOSITES

**Figure 3:**
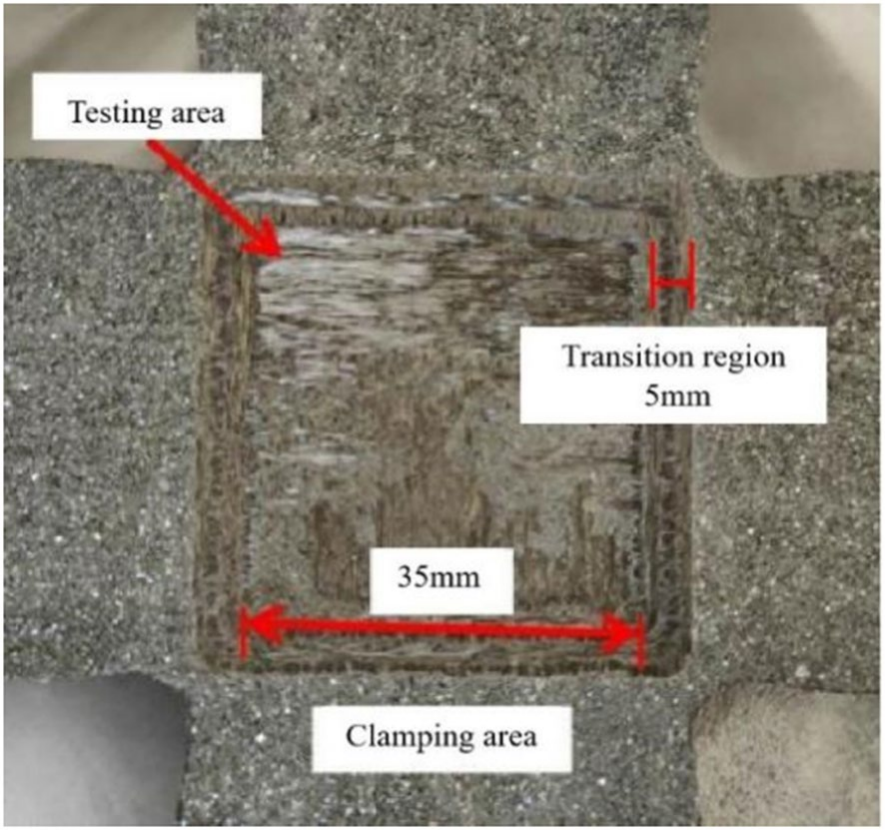
C/C specimen and fixture.

In the present experiments, the heating object was a needle-shaped C/C composite material, with the experimental part structured as a cross shape. TORAY T700-12 K carbon fiber, short cut fiber mesh and weft free fabric were alternately laid (weft free fabric 0°/90° cycle) to prepare needle punched preforms. The density of the prefabricated body prepared using 80 g/m^2^ mesh tire, 380 g/m^2^ weft free fabric, needle depth of 12 mm, and needle density of 22 needles/cm^2^ process parameters reached 0.6 g/cm^3^. The mature asphalt high-pressure immersion carbonization densification process was used to obtain needle-shaped C/C composite material. The laser was positioned within the square area at the centre of the cross shape, measuring 35 mm × 35 mm × 1.5 mm. This central square area was connected to four loading arms, each with a length of 150 mm and a thickness of 4 mm. A transition region of 5 mm was designated between the loading arm and the central experimental area, as depicted in Fig. 3.

**Figure 4:**
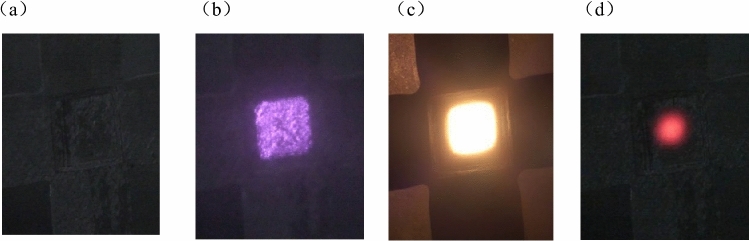
The heating process at the centre of the specimen recorded by the camera under laser irradiation: (**a**) before irradiation; (**b**) 0.04 s; (**c**) heating from 10 s to 1800 °C; and (**d**) end of heating.

Figure 4 shows the heating process of the centre of the specimen recorded by the camera under laser irradiation. During the experiment, the specimen remained in an air atmosphere throughout. An observation can be made that during laser irradiation onto the specimen surface, the camera distinctly captured a square-shaped light spot with a uniform distribution of the laser spot. With the increase in laser irradiation time, the surface temperature of the specimen increased gradually. This resulted in the material surface becoming increasingly bright, and a clear boundary emerged between the area being heated by the laser and the surrounding non-heated region. After laser heating for about 10 s, the temperature of the specimen reached about 1800 °C, and the surface thermal radiation of the specimen was intense. When the laser was stopped, the bright light on the surface of the specimen rapidly decayed back to the shape before the heating began.

**Figure 5:**
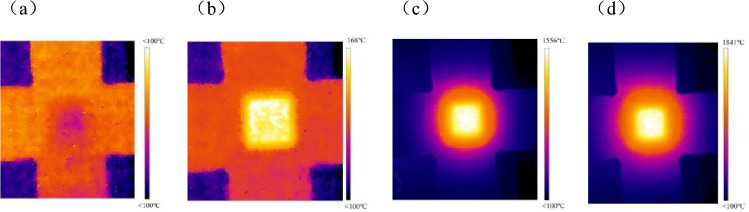
Temperature rise process of material recorded by thermal imager (**a**) before heating; (**b**) laser irradiation 0.033 s; (**c**) laser heating temperature rise process; (**d**) reach the highest temperature of heating.

Figure 5 shows the heating process of the specimen centre under laser irradiation recorded by the infrared thermal imager. During laser irradiation onto the surface of the specimen, heat conduction from the central heating region to the gripper arms became increasingly apparent with the passage of irradiation time. As the laser irradiation continued, there was a gradual increase in temperature along the midline of the specimen’s surface, with the highest temperature reaching approximately 1800 °C. An observation can be made from the thermal dynamic diagram of the infrared thermal imager that the temperature at the edge of laser heating was lower than that at the centre. In the 10 mm × 10 mm area at the centre of the laser heating area, when the highest temperature was 1841 °C, the lowest temperature was 1780 °C, and the temperature difference was 61 °C. As such, in the subsequent analysis of laser heating temperature, the temperature at the centre of the laser heating region was utilised as the characteristic temperature for laser heating.

**Figure 6:**
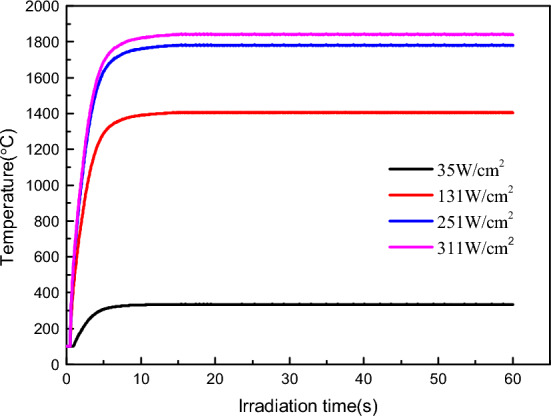
Surface temperature rise process of materials with different laser power densities.

Figure 6 shows the temperature rise process of the material surface under various laser output power densities. The temperature depicted in the image represents the highest temperature recorded at the centre of the specimen by the thermal imager. In the experiment, the power of laser heating was constant. An observation can be made that the temperature rise on the surface of the material was gradually balanced with the increase in irradiation time under laser irradiation with constant power density. Higher laser heating power densities result in higher initial temperature rises of the material and higher final equilibrium temperatures. The temperature rise curve in the diagram illustrates that different equilibrium temperatures of the material could be maintained by controlling the output power of the laser. Thus, by adjusting the laser output power, it is possible to heat the specimen in an aerobic environment and maintain it at different temperatures.

### Short-term thermal shock cycling experiment in aerobic environment

**Figure 7:**
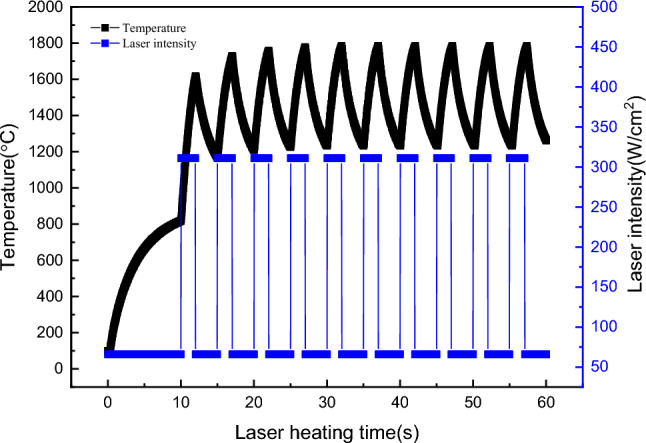
Short-term thermal shock of simulated 245 W/cm^2^ laser at 800 °C.

Since the fibre laser employed in the proposed system allows for easy adjustment of light power through current modulation and offers precise time control down to milliseconds, it was feasible to simulate thermal shock on C/C composite specimens by manipulating the laser output power. Figure 7 presents the experimental outcomes. In the experiment, the laser first output the power density of 66 W/cm^2^. After heating for 10 s, the temperature was basically kept at an equilibrium temperature of about 800 °C. From the previous experiments, an observation can be made that maintaining the laser power density at the aforementioned level resulted in the material’s surface temperature stabilising at approximately 800 °C. The laser heating power density was subsequently adjusted to 311 W/cm^2^, irradiating for 2 s. This abrupt change in laser power density could be divided into two components. Specifically, 66 W/cm^2^ was allocated to maintain the material at 800 °C, while the remaining 245 W/cm^2^ was dedicated to simulating the short-duration laser thermal shock. Therefore, the short-time laser thermal shock with a simulated output power of about 245 W/cm^2^ at 800 °C was equivalent to that at the above power density. The laser output power was then restored to 66 W/cm^2^ and maintained at 3 s. The laser output power density was recirculated between 311 and 66 W/cm^2^ until the heating time reached 1 min. From the analysis of the heating effect, the final heating temperature of the specimen was about 1230–1780 °C during the process of laser repeated heating. The heating method described can effectively simulate both instantaneous laser intensity irradiating the surface shocks at high temperatures and thermal fatigue cycles occurring at high temperatures.

**Figure 8:**
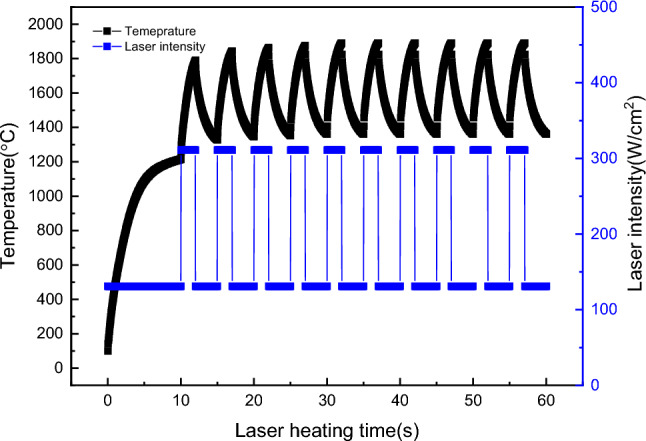
Short-term thermal shock simulation of laser with power density of 180 W/cm^2^ at 1210 °C.

Enhancing the initial laser power density enabled the simulation of short-duration thermal shocks in various high-temperature environments. Figure 8 depicts a scenario simulating a high thermal transient impact at 1210 °C with a power density of 180 W/cm^2^. In the experiment, the laser initially output a power density of 131 W/cm^2^. After heating for 10 s, the temperature stabilised at approximately 1210 °C. Subsequently, the laser heating power density was increased to 311 W/cm^2^ for 2 s before reverting to 113 W/cm^2^ for 3 s. This sequence effectively replicated an instantaneous laser impact laser intensity irradiating the surface of 180 W/cm^2^ at 1210 °C. The laser heating power density was then changed to 311 W/cm^2^, irradiating for 2 s. Subsequently, the laser power density was restored to 113 W/cm^2^ for 3 s. This sequence effectively reproduced a laser instantaneous impact laser intensity irradiating the surface of 180 W/cm^2^ at 1210 °C. The process was iterated until the heating time reached 1 min. From the analysis of the heating effect, it can be observed that the final heating temperature of the specimen ranged from about 1350 to 1870 °C during the repeated laser heating process. This heating method can effectively simulate both instantaneous laser intensity irradiating the surface shocks at high temperatures and thermal fatigue cycles at high temperatures.

### C/SiC control experiment

**Figure 9:**
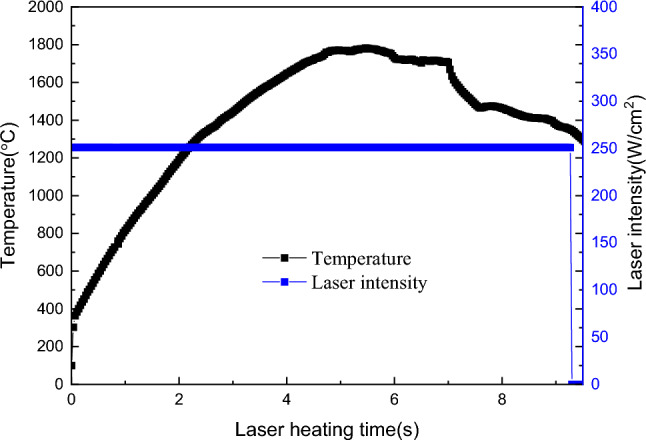
Temperature rise process of C/SiC composites under laser heating (251 W/cm^2^).

In order to verify the universality of high temperature thermal test for common materials, C/SiC composites were fabricated according to the same specimen specification. The experiments of C/SiC composites irradiated by constant laser intensity irradiating the surface laser were conducted under the same experimental conditions. In the experiment, the laser heating power density was 251 W/cm^2^. Figure 9 shows the surface temperature of the specimen recorded by the infrared thermal imager. The results indicate that the temperature rise pattern of C/SiC material resembles that of C/C material during the initial stages of laser heating. However, upon reaching 1800 °C, the temperature began to exhibit significant fluctuations and notably decreased with prolonged laser heating time. Evidently, the objective of maintaining a constant temperature could not be achieved through laser heating with a constant power density.

## Analysis of experimental results

**Figure 10:**
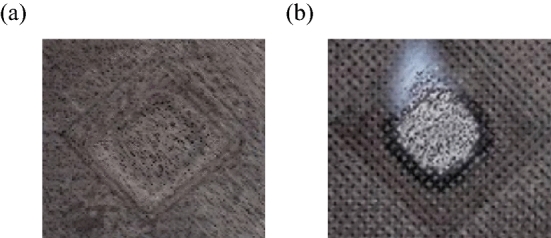
Surface morphology of specimens heated by laser irradiation (**a**) C/C composites; (**b**) C/SiC composites.

Figure 10 shows the surface morphology of C/C and C/SiC composites after laser irradiation. An observation can be made that there were obvious ablation pits on the surface of C/C composites after laser heating. For C/SiC composites, there was no obvious ablation mark after laser heating, but there was obvious white spots at the centre of the specimen surface, which could be attributed to the obvious change in thermal coupling coefficient.

**Figure 11:**
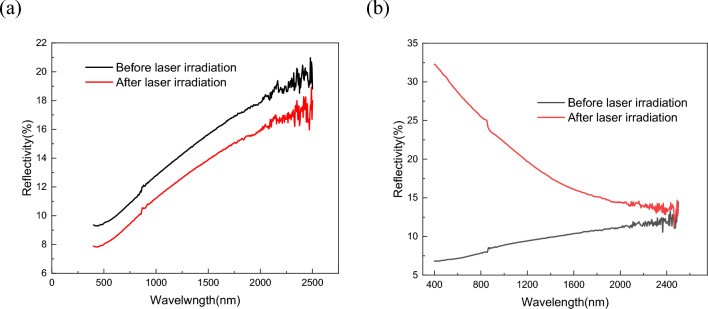
Laser energy coupling coefficient of materials before and after laser heating: (**a**) C/C composites; (**b**) C/SiC composites.

The laser energy coupling coefficient of material surface before and after laser heating was measured by spectrophotometer. The spot illuminated by the spectrophotometer was a long strip with a size of 15 mm × 5 mm, which was located at the center of the laser heating area during testing. Figure 11 shows the measurement results of the surface reflectivity of the material. The test wavelength ranged from 400 to 2500 nm. The transverse coordinates were the wavelength of the laser and the longitudinal coordinates were the reflectivity of the laser. The results show that for C/C composites, the laser reflectivity still increased with the increase in laser wavelength after laser heating to 1850 °C. However, for C/SiC composites, there was a noticeable change in the reflectivity of the specimens after laser heating, which varied significantly with changes in wavelength. Prior to laser heating, the reflectivity of the material increased as the laser wavelength increased. However, following laser irradiation, the reflectivity decreased as the laser wavelength increased. Notably, in the short wavelength direction, the laser reflectivity of the material was considerably higher than in the long wavelength direction.

Given that the absorption depth of the laser was significantly less than the thickness of the material, it was reasonable to conclude that the laser irradiation onto the material surface did not penetrate the material. The incident energy subtracted by the reflected energy, which is the energy that the material converted into heat after absorbing the laser. For C/C composites, the reflectivity of the material to the laser decreased from 13.27 to 11.70% under 1080 nm laser heating, and the absorptivity increased from 86.73 to 88.30% correspondingly. The change rate was about 1.8%. Thus, it could be approximately considered that the laser absorption rate of C/C composites was basically constant when heated by 1080 nm laser. However, for C/SiC materials, the reflectivity changed from 9.14 to 21.19% at the 1080 nm wavelength of the heating laser, the corresponding coupling coefficient of laser energy changed from 90.86 to 68.81%, and the variation rate of laser energy coupling coefficient reached 24.27%. Consequently, a substantial portion of the laser heating energy was reflected off the material surface. This phenomenon may explain why the temperature on the surface of C/SiC material fluctuated under the same laser power density, as shown in Fig. 10. Hence, when there was a sudden change in the laser energy coupling coefficient on the material surface, it became unrealistic to maintain a constant laser intensity irradiating the surface on the material surface simply by using a laser beam with a constant power output. Instead, it became necessary to adjust the output power in real-time according to changes in the surface coupling coefficient to maintain a constant laser intensity irradiating the surface on the material surface.

**Figure 12:**
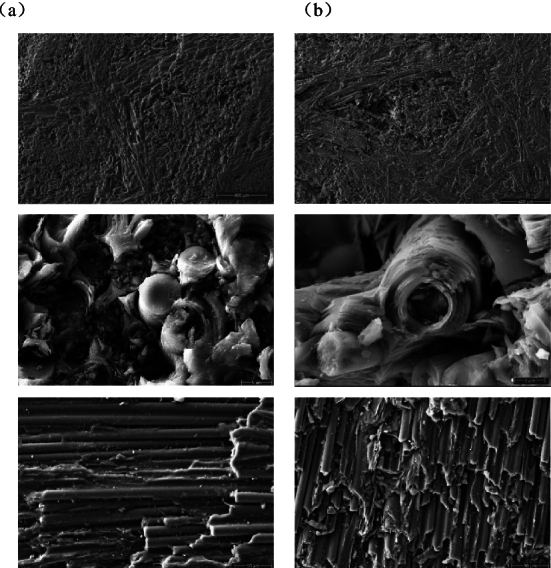
SEM images of the fracture surface of C/C after laser irradiation:(**a**) before laser irradiation, (**b**) after laser irradiation.

In order to further investigate the mechanism and influencing factors of laser energy coupling coefficient mutation, scanning electron microscopy (SEM) analysis was conducted on C/C composites before and after laser heating, as shown in Fig. 12. The results show that under the action of high laser intensity irradiating the surface and oxidation by laser heating, the inner fibre surface of C/C composites was ablative, and the SEM images show obvious ablative pits. Upon observing the damage to carbon fibres on a smaller scale, it was noted that in the absence of laser heating, the section of carbon fibre filament was neat. Nevertheless, after laser heating, the length of carbon fibre filament was different and there were a large number of fragments around it. The analysis indicates that the high temperatures induced by laser irradiation resulted in significant mass loss of carbon fibre due to high-temperature oxidation. Subsequently, the sublimation of certain carbon fibres contributed to incomplete fibre filaments and noticeable breakage of carbon fibre filaments and matrix.

**Figure 13:**
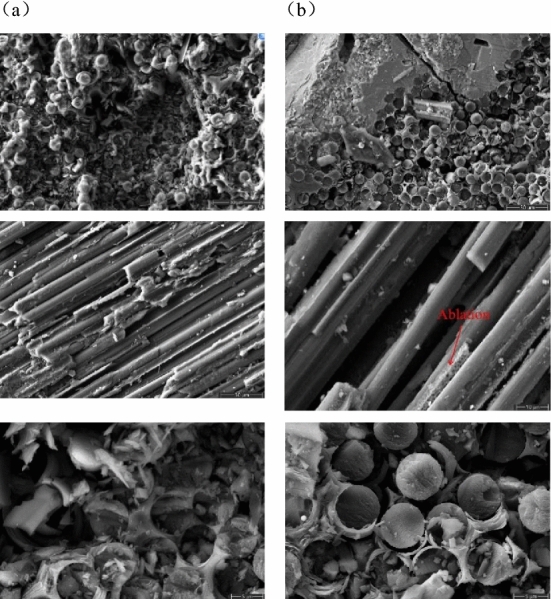
SEM images of the fracture surface of C/SiC after laser irradiation:(**a**) before laser irradiation, (**b**) after laser irradiation.

The central position of C/SiC specimen before and after laser heating was observed using SEM. The observed results are shown in Fig. 13. The results show that the carbon matrix of the specimen can be observed after laser heating. It was hypothesised that this phenomenon could be attributed to the presence of an aerobic environment, leading to the oxidation of materials in the air.

**Figure 14:**
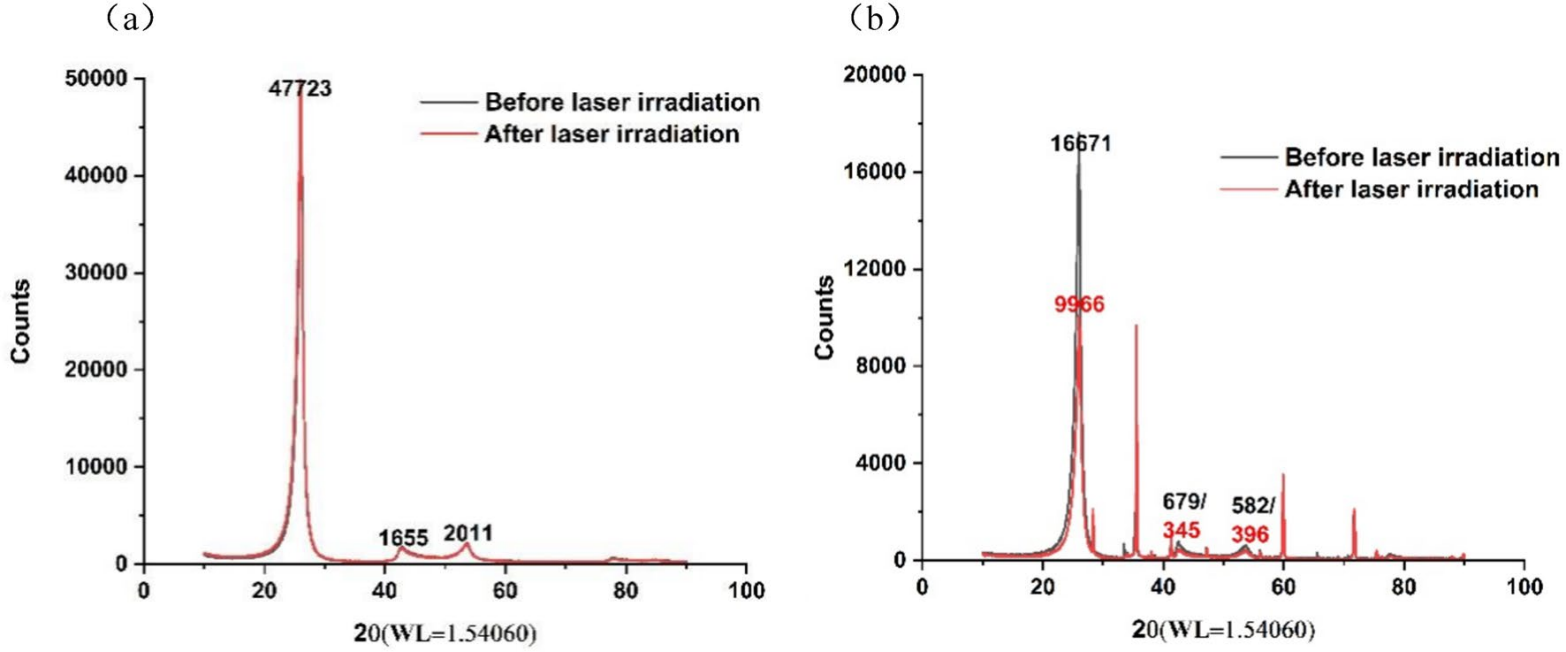
XRD spectra of C/SiC composites: (**a**) C/C, (**b**) C/SiC.

**Figure 15:**
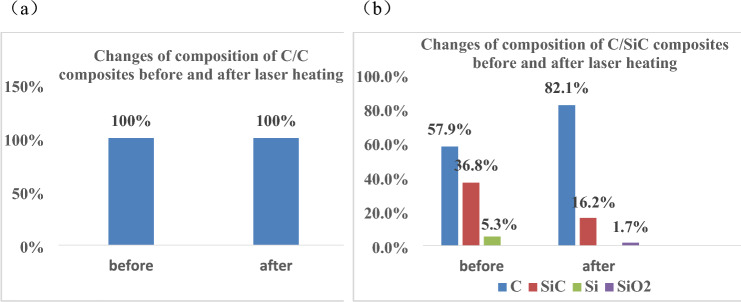
The composition changes of the two composites before and after laser heating: (**a**) C/C, (**b**) C/SiC.

The composition changes of the two materials before and after laser heating were calculated by means of XRD spectra as shown in Figs. 14 and 15.

An observation can be made that the main component C in C/C composites did not change before and after laser heating. However, the composition and C of C/SiC changed from 57.9 and 36.8 to 82.1% and 16.2%, respectively, before and after laser heating. Notably, SiO_2_ components were present in the materials after laser heating, accounting for 1.7%, which could be ascribed to the chemical reactions of C/SiC materials under aerobic and high temperature conditions. The reaction equation is shown as follows,

$$ {\text{SiC }} + {\text{ O}}_{{2}} \to {\text{ SiO}}_{{2}} + {\text{ C}} $$

It can be seen that the above-mentioned chemical reaction process of SiC in high temperature aerobic environment forms carbon products, resulting in an increase in carbon content after laser irradiation. Meanwhile, it is precisely due to the formation of SiO2 and C products in C/SiC materials induced by laser irradiation that the coupling coefficient and reflectivity of the materials were altered, leading to changes in the temperature of the material surface.

## Conclusion

In the present study, the feasibility of applying laser heating to ultra-high temperature thermal simulation experiments of high temperature resistant materials was explored. It was concluded that the utilisation of high homogeneity square flat-top laser beams demonstrated exceptional applicability in thermal simulation experiments involving high-temperature resistant materials in an aerobic environment. This method enables the simulation of high laser intensity irradiating the surface heating and rapid temperature rise rates, which cannot be achieved by traditional heating methods. In the present study, the output of a flat-topped laser beam with high homogeneity was achieved through C fibre bunching, while laser output power density was controlled via current modulation. During heating, C/C composites were rapidly heated to approximately 1850 °C within minutes, with the highest temperature rise rate reaching 500 °C/s. Further, the system exhibited the capability to conduct thermal fatigue cycle tests by controlling the laser current. Overall, the proposed experimental system and method provide a novel approach for the thermal simulation investigation of high-temperature resistant materials.

The application scope of the experimental technology was also further investigated. The experimental results of laser heating of C/SiC composites show that, unlike laser heating of C/C composites, when C/SiC specimens are heated by laser in an aerobic environment to about 1200 °C, the surface temperature of the specimens began to decrease under the heating of constant laser power density. This phenomenon occurred due to the formation of SiO_2_ crystals on the surface of SiC material under the combined influence of aerobic conditions and high temperatures. These crystals exhibited strong reflectivity to the heating laser, resulting in a reduction of laser energy effectively converted into heat. This analysis highlights the significance of the laser absorption coefficient of the material in determining the effectiveness of the method. When the laser absorption coefficient of the material abruptly changes due to the generation of new products, simulating constant laser intensity irradiating the surface through constant laser heating power may lead to significant errors. In such cases, adjusting the laser output power according to the surface state of the material becomes necessary to realise specific temperature rise processes, thereby imposing higher control requirements on the heating system. These findings suggest that further research into the experimental system and methods is warranted.

## Data Availability

The datasets used and analysed during the current study available from the corresponding author on reasonable request.
